# Dihydrogen Bond in the Aminoborane Complex of a Nicergoline Intermediate

**DOI:** 10.3390/molecules24142548

**Published:** 2019-07-12

**Authors:** Jan Čejka, Ladislav Cvak, Simona Žižková, Bohumil Kratochvíl, Alexandr Jegorov

**Affiliations:** 1 Department of Solid State Chemistry, University of Chemistry and Technology Prague, Technická 5, 166 28 Prague 6, Czech Republic; 2 Teva Czech Industries s.r.o., Research and Development, Ostravská 29, 747 70 Opava-Komárov, Czech Republic; 3 Teva Czech Industries s.r.o., Research and Development, Branišovská 31, 370 05 České Budějovice, Czech Republic

**Keywords:** nicergoline, aminoborane, CSD, single crystal, X-ray structure, dihydrogen bond

## Abstract

An aminoborane side product from the nicergoline manufacture process was identified by single-crystal X-ray diffraction. As boranes of pharmaceutical molecules are quite rare, the binding potential of the BH_3_ group was investigated and compared with similar compounds using Cambridge Structural Database (CSD). Surprisingly, the packing was stabilized by a dihydrogen bond, which triggered a false alert for too-short contact of hydrogen atoms in IUCR checkCIF. As the dihydrogen bond concept is not widely known, such an alert might mislead crystallographers to force –CH_3_ optimal geometry to –BH_3_ groups. The B–H distances equal to or less than 1.0 Å (17% of the CSD structures) are substantially biased when analyzing the structures of aminoborane complexes in CSD. To conduct proper searching, B–H bond length normalization should be applied in the CSD search.

## 1. Introduction

Sodium borohydride is widely used as a reducing agent in the synthesis of many active pharmaceutical substances. By contrast, the possibility that a reaction could be accompanied by the formation of a boron-containing side product is not usually considered. In the course of the reaction of daunomycin with sodium borohydride, various borate esters were observed. In this case, the coordination of boron to the drug proceeded via an oxygen atom [1]. For a number of other sodium borohydride-mediated reactions, the formation of borate esters was also observed [2].

Aminoboranes [3] are usually known as hydrogenation agents. They offer some advantages over sodium borohydride, such as good solubility in organic solvents. They are less sensitive to acids and can serve as chiral reducing agents [4,5,6]. On the other hand, they are generally weaker reducing agents. Despite their popularity, very few crystal structures of boranes of alkaloids or pharmaceutical molecules have been described. As a rare example, the crystal structures of several borane complexes of cinchona alkaloids have been reported [7].

**Figure 1:**
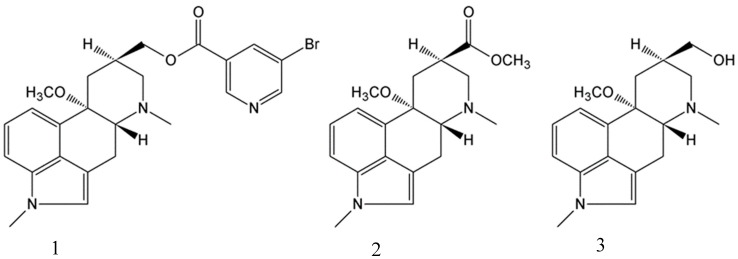
Scheme of nicergoline (**1**), ester (**2**), and alcohol (**3**-MeLuol) intermediates.

During the years 1984 and 1985, we were developing a process for the manufacture of nicergoline (**1**). The process involved the reduction of ester **2** to alcohol **3** (Figure 1). For safety reasons, we avoided the utilization of LiAlH_4_ and developed the reduction with NaBH_4_ in methanol and/or ethanol. During the development of the process, we observed the formation of a lipophilic side product in the reaction mixture. However, the product disappeared during the standard workup: the addition of water, evaporation of methanol and/or ethanol, and isolation of crystalline alcohol **3** from aqueous solution.

After the reduction, when the reaction mixture was worked up by partitioning between dichloromethane and water, the lipophilic side product remained in solution. It was isolated by chromatography of the dichloromethane phase and consequent crystallization from toluene. However, at that time, we were not able to reveal its structure when using NMR and MS. As the impurity was not present in the produced alcohol **3**, we abandoned our pursuit. At that time, the NMR identification failed because the potential presence of boron was not considered, and further attempts were unsuccessful.

**Figure 2:**
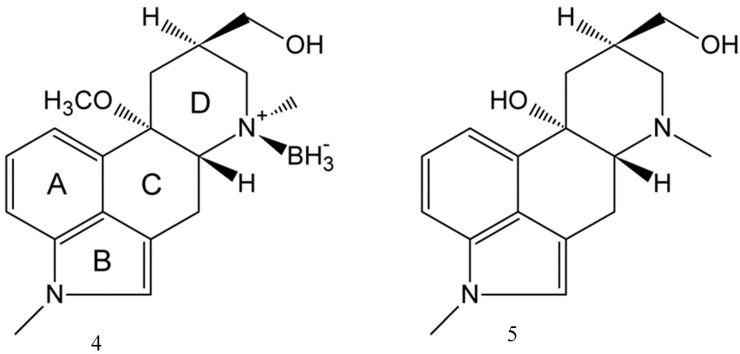
Scheme of BH_3_-MeLuol complex (**4**) and Luol (**5**).

**Figure 3:**
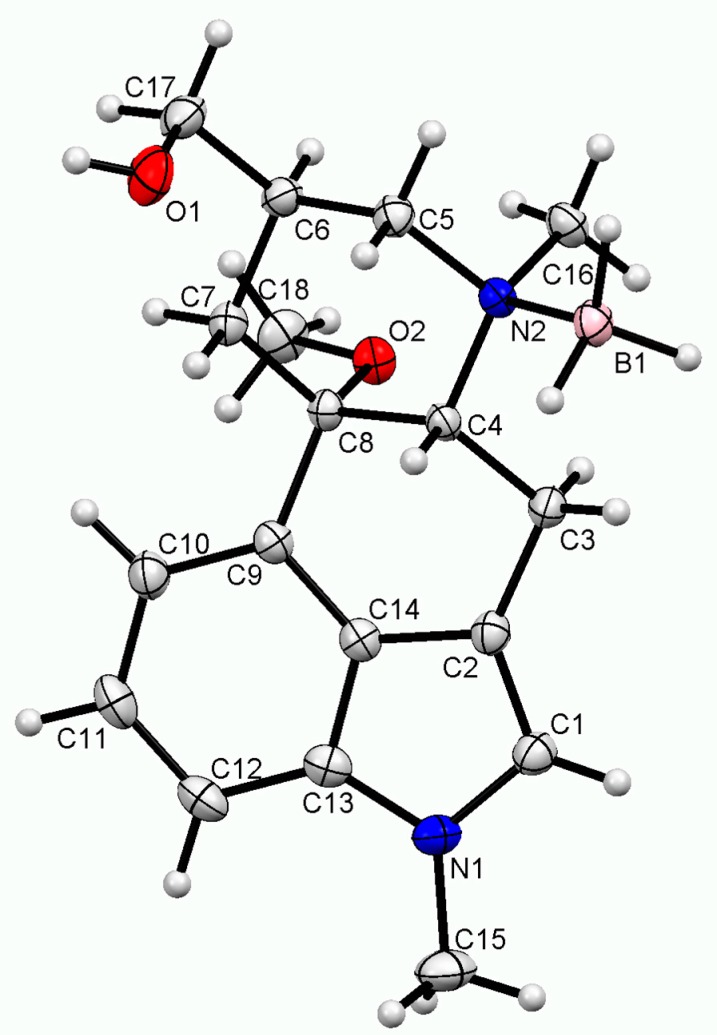
ORTEP drawing of BH_3_-MeLuol (**4**) with atomic numbering, ADP drawn at 50% probability level.

We have thus reinvestigated this side product and decided to determine its structure using X-ray diffraction. X-ray structure determination was chosen because of “*its power to show totally unexpected and surprising structure with, at the same time, complete certainty*” [8]. And indeed, structure determination revealed the unexpected presence of boron, Figure 2 and Figure 3.

### Dihydrogen Bond

The dihydrogen bond is a relatively new phenomenon among weak intermolecular interactions. It is a non-conventional hydrogen bond, described by Crabtree et al. in 1996 as “*H–H or dihydrogen bond between a conventional hydrogen bond donor such as an NH or OH group as the weak acid component and an element-hydride bond as the weak base component, where the element in question can be a transition metal or boron*” [9]. It is rarely mentioned in crystallography textbooks [10]. For a complete review of the dihydrogen bonding concept, see [11].

## 2. Results and Discussion

### 2.1. Preparation of Single Crystals

Powder BH_3_-MeLuol (**4)** (74 mg) was dissolved in ethyl formate (7 mL). The solution was allowed to slowly evaporate in open vial at 298 K. Single crystals were formed within two days after the volume of the solution was reduced to about two-thirds.

### 2.2. Single-Crystal X-ray Diffraction Structure

BH_3_-MeLuol (**4;** borate(1-), trihydro-*N*6-(1methyl-10α-methoxy-9,10-dihydrolysergol(1+))) crystallizes in the orthorhombic system, space group *P* 2_1_ 2_1_ 2_1_, one molecule in the asymmetric part of the unit cell. Full crystallographic details are given in Table 1. Please note that the systematic ergoline numbering *N*6 corresponds to *N*2 in the current crystal structure).

### 2.3. Impact of BH_3_ on the MeLuol Conformation

**Figure 4:**
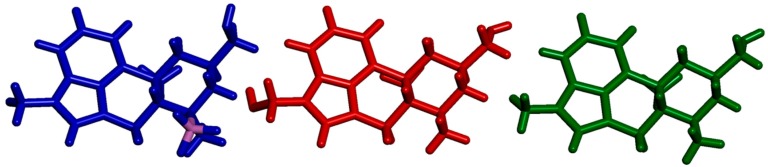
Parallel-oriented molecules of Luol derivatives. BH_3_-MeLuol (**4**)—blue, Luol (**5**)—red, MeLuol (**3**)—green, boron—pink.

The side product **4** is a derivative of MeLuol (**3**; 1-methyl-10α-methoxy-9,10-dihydrolysergol). The complexation of **3** with borane had a very low influence on the original molecular conformation of **3** [12,13]. An LSQ fit of the molecules was calculated in DSViewer [14] to provide an illustrative overview (Figure 4) of the particular conformations. 

**Figure 5:**
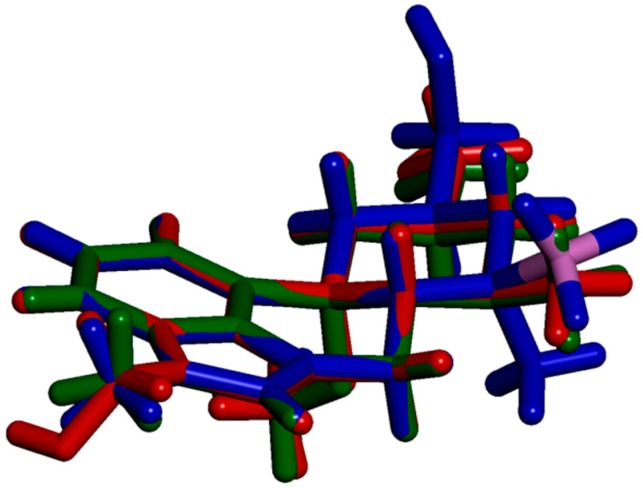
Side-oriented fitted molecules of Luol derivatives. BH_3_-MeLuol (**4)**—blue, Luol (**5**)—red, MeLuol (**3**)—green, boron—pink.

There are three subtle differences in the molecular conformations. The hydroxymethyl group of **4** is rotated to the torsion angle of C5–C6–C17–O1= −55.54(14)°. The corresponding dihedral angles are C7–C8–C20–O21 = 165.49(18)° in Luol (**5** = 1-methyl-10α-hydroxy-9,10-dihydrolysergol) and C7–C8–C19–O20 = 166.29(11)° for MeLuol (**3**). The conformational change is promoted by the change of molecular packing as the hydroxyl group binds to the same location occupied by borane in **4** and the lone pair of the tertiary amine in **3** and **5**. In **4**, the methyl adjacent to *N*2 amine adopts position switched to below plane, while its site is occupied by borane. Another difference can be found in the conformation of ring D (see Figure 2 for the ring system lettering), which is shallower in **4** compared to **3** and **5** (Figure 5). The respective puckering amplitudes [15] of the chair conformations represent quantifications of the visual differences: 0.5359(14) Å for **4**, 0.578(2) Å for **5**, and 0.5792(11) Å for **3**.

### 2.4. Structural Features

#### 2.4.1. Molecular Packing Compared to Related Derivatives

The positions and isotropic ADP’s hydrogen atoms bonded to hetero atoms B and O were refined with soft restraints. 

The molecular packing and conformation were compared with related structures of **3** and **5** (CCDC code: RAXPEM, JAZPAC) [12,13].

The molecules of **4** formed infinite zigzag chains via O–H … H_2_-(HB) bifurcated dihydrogen bonds along the *c*-axis. The fused rings of two adjacent molecules form an angle of 51°. The packing was stabilized by weak C–H … π contacts.

In **5**, the molecules form similar infinite zigzag chains along *a*-axis using O–H … N hydrogen bonds. Due to less space, the adjacent molecules are more tilted. They bind to each other in an almost perfect perpendicular arrangement. The angle between the fused rings of the neighboring molecules is 87°. The chains are crosslinked by an O–H … O hydrogen bond to the first O–H group.

The molecules of **3** form virtually the same infinite zigzag chains along the *a*-axis by employing O–H … N hydrogen bonds. As in **5**, due to less space, the adjacent molecules are more tilted. They bind to each other in an almost perfect perpendicular arrangement. The angle between the fused rings of the neighboring molecules is 88°. As there are no more hydrogen bond donors, the infinite rings are crosslinked, and crystal packing stabilized by weak C–H … O improper hydrogen bonds.

#### 2.4.2. Biased B–H Bonds in CSD

There are almost no structures of pharmaceutically used aminoboranes. As the dihydrogen bond is a relatively new phenomenon [9,10,11,16], more effort was invested in the investigation of binding features of the borane group intermolecular binding ability.

The coordination of borane in **4** was compared to X-ray structures of similar BH_3_-*N*-methyl-cyclohexane-containing structures (CCDC code: XANFUN, QOPGOR) [17,18]. For the list of BH_3_-*N* geometry, see Table 2. Under the brief inspection of the CIF data, it was clear that the B–H bond length distances of the published structures (XANFUN, QOPGOR) were set according to sp^3^ geometry, but the B–H distances were erroneously fixed as equal to C–H bond distances. Interestingly, the CSD (May 2019) contains 488 structures with BH_3_-*N* aminoboranes, when neglecting no-hydrogen and no-coordinate deposits. In 114 structures, all B–H bond lengths were smaller than 1.1 Å. The *d*(B–H) values are 1.0 Å or less for all B–H bonds in 86 structures (23 published during the last five years). The B–H bond length was experimentally determined as 1.190 Å by FTIR [19]. Deviations of the B–H bond length of about 0.05 Å were reported, however, for terminal atoms, was about 0.1 Å [20]. Investigations based on CSD data mining, e.g., [21], are inevitably based on partially biased data, and the authors highlight the inaccuracy in hydrogen atom positions. By contrast, Crabtree et al., in 1996, used normalized B–H and N–H bond distances to avoid systematic underestimation of the bond lengths in the CSD structures [9].

**Figure 6:**
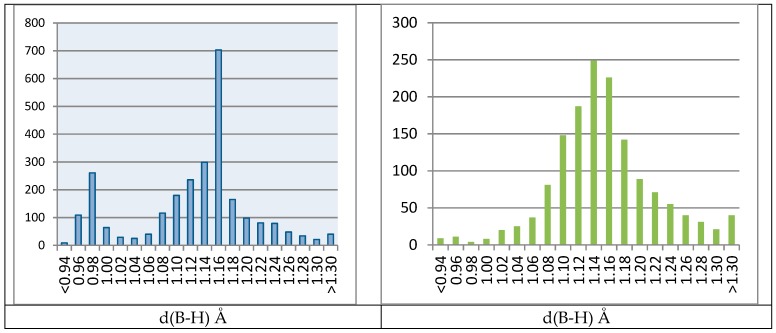
Histograms of bond lengths for all B–H bonds (**left**) and B–H bonds in probably refined BH_3_ groups (**right**).

Structures containing *N*-BH_3_ groups were extracted from CSD. The observations are summarized in histograms of B–H bond lengths in *N*-BH_3_ groups giving a statistical overview of 2637 B–H bonds (879 BH_3_ groups) in 488 CSD published structures (Figure 6). Using simple criteria, the data were split to two groups: structures in which position of the BH_3_ hydrogen atoms were probably refined (1494 bonds in 318 structures), and structures with B–H bond distances set according to the optimal geometry or forced by strong restraints (1143 bonds in 170 structures). As the CSD does not contain standard uncertainties, the criterion—whether the BH_3_ hydrogen atoms were refined—was based on the difference of min. and max. *d*(B–H) of each BH_3_ group. In general, non-refined or firmly restrained BH_3_ groups should have zero or very low variances of *d*(B–H). The criterion was set to 0.01 Å in the middle of the range of acceptable values, estimated from 0.000 to 0.020 Å. The histogram of all B–H bond lengths clearly showed two distinct statistical distributions with two strong maxima at 0.96–0.98 Å and 1.14–1.16 Å. On the other hand, the histogram of the B–H bond lengths for the group of structures with likely refined BH_3_ groups had only one broad maximum at 1.14–1.16 Å, and fairly low remnants of 0.96–0.98 Å. The average value of B–H bond length of 1.118 Å for all structures was far from false values, those below 1.00 Å. When calculated from 318 likely refined structures, the average value was 1.144 Å. It should be noted that all non-refined BH3 groups were affected.

Unfortunately, the problem is likely connected to (415_ALERT_2_B Short Inter D-H..H-X) in the checkCIF/Platon IUCR validating tool [22]. “*Obviously, when atoms approach closer than the sum of their van der Waals radii there must be either a missed interaction, such as a hydrogen bond, or their positions are in some way in error*” [23]. On the other hand, the Platon software, which alerts for close contact, properly puts the dihydrogen bond on the list of the hydrogen bonds (CALC ALL procedure). Some authors, not aware of dihydrogen bond presence, may restrain or fix *d*(B–H) in the same way and with the same values as for C–H bonds (86 N-BH_3_ groups containing CSD structures). “*Many single-crystal structure analyses are currently carried out by non-experts using the available black-box software*” [22]

The complete list of bond lengths, criterion setting, and statistical analysis is available in Supplementary Materials in the XLSX file.

#### 2.4.3. Impact of BH_3_ on the Molecular Packing

Even when applying normalization of hydrogen atoms in CSD Conquest [24], the XANFUN and QOPGOR packing networks are built only by very weak improper dihydrogen bonds C–H … H. To find structures with hydrogen bond networks similar to **4**, a search was performed for the O–H … H–B–N motif, with *d*(H…H) limited to 2.5 Å. The following structures were found: ABUKAJ [25], DAZPIE [26], DORHAV [27], DUZNUI [28], and WANTAI [29]. In all five structures, the role of BH_3_ in stabilizing the structure via dihydrogen bonds was obvious (Table 3).

Generally, there are only small variations of structure packing. The hydrogen bond to BH_3_ is either single (DORHAV, DUZNUI, WANTAI) or bi-furcated (ABUKAJ, DAZPIE), similarly to the structure of **4**.

## 3. Materials and Methods

### 3.1. Single-Crystal X-ray Diffraction

Single-crystal X-ray crystallographic diffraction data were collected at 180 K using a Bruker D8 Venture diffractometer equipped with Cu Kα radiation (*λ* = 1.54178 Å), polycapillary monochromator, Photon 100 detector (Bruker ASX Inc., Madison, WI, USA). The habitus of the crystals could be described as parallel-oriented aggregates of pillars. The diffraction quality of the vast majority of the crystals was affected, producing diffused elongated diffraction spots that quickly lost diffraction power. Finally, a single crystal of outstanding quality was found, dipped in paraben oil, fixed to a 100 μm MicroMount mounted to the goniometer head, and immediately cooled to 180 K for data collection. The data collection, indexing of reflections, determination of the unit cell parameters, integration of the intensity of the reflections, and frame scaling were performed using the Bruker Saint software [30]. The structure was solved by direct methods with SIR92 [31] and refined with CRYSTALS [32]. Geometry calculations including puckering parameters were conducted in Platon [22]. Molecular visualization was provided by Mercury [33] and DS Viewer [14]. The positional and atomic displacement parameters of all non-hydrogen atoms were refined. All H atoms were located in a difference map, but repositioned geometrically, and were then initially refined with soft restraints on the bond lengths and angles to regularize their geometry (C–H in the range of 0.93–0.98 Å, B–H to 1.15 Å, and O–H to 0.82 Å) and *U_iso_*(H) (in the range 1.2–1.5 times *U_eq_* of the parent atom), after which the positions of carbon-bound hydrogen atoms were refined with riding constraints. The same set of soft restraints was used during the final stages of the refinement when the positional parameters of the hetero bound hydrogen atoms were refined. The absolute configuration was resolved by the refinement of Flack *x* = 0.06(18) and the calculation of Hooft *y* = 0.00(7) parameters [34]. The chiral centers were assigned as C4 *R*, N2 *S*, C6 *R*, and C8 *R*. The C8 configuration was calculated incorrectly by Platon software. The configuration of the chiral centers was in agreement with expected chirality. No additional solvent-accessible voids were found in the structure. The crystallographic data are summarized in Table 2. 

CCDC 1919106 contains the supplementary crystallographic data for this paper. These data can be obtained free of charge via http://www.ccdc.cam.ac.uk/conts/retrieving.html (or from the CCDC, 12 Union Road, Cambridge CB2 1EZ, UK; Fax: +44 1223 336033; E-mail: deposit@ccdc.cam.ac.uk)

### 3.2. Materials Used

The historical source of **3**: Galena Co., Opava, Czech Republic (Teva Pharmaceutical Industries Ltd., Opava, Czech Republic) Solvents were obtained from commercial sources (Sigma Aldrich, Prague, Czech Republic). 

MicroMount 100 μm, M1-L19-S2, MiTeGen, Ithaca, NY, USA.

Parabar 10312, HR2-643, Hampton Research, Irvine, CA, USA.

## 4. Conclusions

We have crystallized and solved the crystal structure of BH3-MeLuol with a remarkable hydrogen bond network featuring a dihydrogen bond. When comparing the molecular packing to the published CSD structures, we have encountered a problem with a significant occurrence of suspiciously short B-H bond lengths in the CSD. The values resemble methyl optimal geometry. We have demonstrated the statistical relation of the false values with the absence of hydrogen atoms refinement. Surprisingly, the B–H average distance, calculated from all B–H bonds, was only slightly biased. The B–H intermolecular contacts are up to 0.2 Å longer in almost 18% of the published structures, which affects statistical studies dealing with weak inter- and intramolecular interactions. A CSD user who is aware of the problem can handle it with an optional normalization of B–H lengths to at least 1.15 Å as available in the Conquest search. As the source of the bias is human error, we would like to emphasize this problem. The structures containing dihydrogen bonds trigger false alerts in IUCR checkCIF (based on Platon software [22]) on short contacts between hydrogen atoms (*415_ALERT_2_B Short Inter D-H..H-X*). Some authors, not aware of the presence of the dihydrogen bond, may restrain or fix the B–H distances in the same way and using the same values as for C–H bonds. 

We have found a group of N-BH3 containing structures in the CSD with a similar hydrogen bond network. Sometimes, likely due to its good accessibility the BH_3_ group can compete with the available conventional hydrogen bond acceptors.

## Figures and Tables

**Table 1:** Crystallographic data of the structures.

Empirical formula	C_18_H_27_BN_2_O_2_
Formula weight	314.24 g/mol
Temperature	180 K
Wavelength	1.54178 Å
Crystal system	Orthorhombic
Space group	*P* 2_1_ 2_1_ 2_1_
Unit cell dimensions	*a* = 9.6759(14) Å
	*b* = 11.8394(16) Å
	*c* = 14.752(3) Å
*V*	1690.0(7) Å^3^
*Z*	4
Density (calculated)	1.235 g/cm^3^
Absorption coefficient	0.623 mm^−1^
*F_000_*	680.0
Crystal size	0.152 × 0.280 × 0.408 mm^3^
Theta range for data collection	4.79° to 72.147°
Index ranges	−11 ≤ *h* ≤ 11, −14 ≤ *k* ≤ 14, −17 ≤ *l* ≤ 18
Reflections collected	20314
Independent reflections	3301 [*R_int_* = 0.036]
Completeness to *θ* = 72.147°	99.999%
Max. and min. transmission	0.75 and 0.91
Refinement method	Full-matrix least-squares on *F*^2^
Data/restraints/parameters	3301/5/226
Goodness-of-fit on *F^2^*	1.001
Final *R* indices [*I* > 2σ(*I*)]	*R*_1_ = 0.0311, *wR*_2_ = 0.0731
*R* indices (all data)	*R*_1_ = 0.0296, *wR*_2_ = 0.0700
Extinction coefficient	189(4)
Largest diff. peak and hole	0.19 and −0.15 e·Å^−3^
Flack *x*	0.06(18)

**Table 2:** Aminoborane group comparison with published results.

Compound	*d*(B–N) (Å)	*d*(B–H) (Å)
**4**	1.6358(18)	1.144–1.160(13)
**QOPGOR**	1.608(4)	0.96 *
**XANFUN**	1.621(3)	0.96 *
**ABUKAJ**	1.600(3)	1.154(16)
**DAZPIE**	1.580(6), 1.574(5)	1.057–1.155(8), 0.97–1.21(8)
**DORHAV**	1.606(4)	1.02–1.13(3)
**DUZNUI**	1.627(3)	1.10–1.21(3)
**WANTAI**	1.610(4)	1.14–1.18(2)

* not refined, incorrect distance.

**Table 3:** Dihydrogen bonds connected to BH_3_ in similar compounds.

D–H … A*	D–H (Å)	D … A (Å)	H … A (Å)	D–H … A (°)	D … B (Å)	A…H…A′ (°)
**4**						
O1–H12 … H14	0.826(15)	2.983(17)	2.27(2)	145(2)	3.3911(18)	
O1–H12 … H15	“	2.809(17)	2.04(2)	156(2)	3.3911(18)	51.2(7)
C1–H11 … H13	0.938	3.184(17)	2.398(17)	141.3(3)	3.605(2)	
**ABUKAJ**						
O17–H17O … H18B	0.91(3)	2.881(15)	2.07(4)	148(3)	3.305	
O17–H17O … H18C	“	2.738(11)	1.93(3)	148(3)	“	56.1(10)
**DAZPIE**						
O22–H2 … H1A	0.97	3.00	2.14	147	3.394(9)	
O22–H2 … H1B	“	2.76	2.11	122	“	52
O12–H1 … H2A	1.12	2.890	2.28	112	3.408(8)	
O12–H1 … N21	“	3.460(4)	2.35	171	-	76
**DORHAV**						
O1–H1 … H91	0.78(3)	2.80(3)	2.05(4)	165(3)	3.249(5)	
**DUZNUI**						
O2–H1 … H1B1	0.89(3)	2.62(2)	1.76(4)	162(3)	3.498(3)	
**WANTAI**						
O–H10 … H2B	0.88(3)	2.68(2)	1.81(4)	173(3)	3.264(3)	

* D—hydrogen bond donor, A—hydrogen bond acceptor, H—hydrogen, B—boron. For complete list of proper and improper hydrogen bonds, including ARU codes, see Supplementary Materials.

## Supplementary Materials

The supplementary materials are available online.
