# Supplementary material for: Dihydrogen Bond in the Aminoborane Complex of a Nicergoline Intermediate
Source: Molecules. 2019 Jul 12;24(14):2548. doi: 10.3390/molecules24142548 (PMC6680414; doi:10.3390/molecules24142548)
Supplement: Supplementary file 1 [file molecules-24-02548-s001.pdf]

Dihydrogen Bond in Amine Borane Complex of Nicergoline Intermediate

Jan Čejka[1*], Ladislav Cvak[2], Simona Žižková[1], Bohumil Kratochvíl[1] and Alexandr Jegorov[2,3]

1 Department of Solid State Chemistry, University of Chemistry and Technology Prague, Technická 5, 166 28 Prague 6, Czech Republic; Jan.Cejka@vscht.cz; Simona.Zizkova@vscht.cz; Bohumil.Kratochvil@vscht.cz

2 Teva Czech Industries s.r.o., Research and Development, Ostravská 29, 747 70 Opava - Komárov; Ladislav.Cvak@tevapharm.cz

3 Teva Czech Industries s.r.o., Research and Development, Branišovská 31, 370 05 České Budějovice, Czech Republic; Alexandr.Jegorov@tevapharm.cz

* Correspondence: Jan.Cejka@vscht.cz

SUPPLEMENTARY MATERIAL

Complete list of hydrogen bonds of 4 and the relevant CCDC

4; borate(1-), trihydro-N6-(1methyl-10α-methoxy-9,10-dihydrolysergol(1+)

CCDC 1919106

Donor --- H....Acceptor	ARU	D – H [Å]	H...A [Å]	D...A [Å]	D - H...A [°]	A..H..A* [°]
O(1) --H(12) ..H(14)	2574	0.826(17)	2.27(2)	2.983(16)	144.9(19)	
O(1) --H(12) ..H(15)	2574	0.826(17)	2.04(2)	2.809(15)	156(2)	51.2(7)
C(3) --H(31) ..H(15)		0.97	2.20	2.886(16)	127	
C(4) --H(41) ..H(13)		0.96	2.26	2.674(16)	105	
C(5) --H(51) ..O(1)		0.97	2.44	2.8095(16)	102	
C(7) --H(71) ..O(1)		0.98	2.59	2.9450(18)	102	
C(7) --H(71) ..O(1)		0.95	2.39	2.9761(17)	120	

[2574.] =1/2-x,2-y,-1/2+z

ABUKAJ

http://dx.doi.org/10.1039/b103936a

Donor --- H....Acceptor	ARU	D – H [Å]	H...A [Å]	D...A [Å]	D - H...A [°]	A..H..A* [°]
O(16) --H(16O) ..O(17)	4645	0.88(4)	1.96(4)	2.780(2)	156(3)	
O(17) --H(17O) ..H(18B)	4645	0.91(3)	2.07(4)	2.881(15)	148(3)	
O(17) --H(17O) ..H(18C)	4645	0.91(3)	1.93(3)	2.738(11)	148(3)	56.1(10)
C(16) --H(16) ..O(12)		0.997(16)	2.537(17)	3.049(3)	111.7(10)	
C(26) --H(26) ..O(16)	2665	1.01(2)	2.38(2)	3.214(3)	139.8(10)	

[4645] = 1-x,-1/2+y,1/2-z

[2665] = 3/2-x,1-y,1/2+z

DAZPIE

http://dx.doi.org/10.5517/cc9m1qn

Donor --- H....Acceptor	ARU	D – H [Å]	H...A [Å]	D...A [Å]	D - H...A [°]	A..H..A* [°]
O12 --H1 ..N21	1655	1.12	2.35	3.460(4)	171	
O12 --H1 ..H2A	1655	1.12	2.28	2.89	112	76
O22 --H2 ..H1A	1455	0.97	2.14	3.00	147	
O22 --H2 ..H1B	1455	0.97	2.11	2.76	122	52
C113 --H113 ..H2A	1645	1.03	2.23	3.17	150	
C110 --H110A ..O12		1.04	2.27	3.050(4)	130	
C210 --H210A ..O22		1.04	2.29	3.003(4)	125	
C12 --H12A ..N11		1.04	2.45	2.923(9)	107	
C22 --H22A ..N21		1.04	2.47	2.966(8)	108	
C113 --H113 ..H2A	1645	1.03	2.23	3.17	150	
C120 --H120 ..O22	1555	1.05	2.47	3.455(8)	155	

C217 --H217 ..O12	2755	1.05	2.42	3.220(7)	132	

[1655] = 1+x,y,z

[1645] = 1+x,-1+y,z

[1455] = -1+x,y,z

[2755] = 2-x,1/2+y,-z

DORHAW

http://dx.doi.org/10.5517/cc13g4k8

Donor --- H....Acceptor	ARU	D – H [Å]	H...A [Å]	D...A [Å]	D - H...A [°]	A..H..A* [°]
O(1) --H(1) ..H(91)	1655	0.78(3)	2.05(4)	2.80(3)	165(3)	
N(1) --H(1B) ..O(1)		0.99(3)	2.38(3)	2.872(3)	110(2)	
N(1) --H(1B) ..O(1)	2567	0.99(3)	2.06(3)	2.935(4)	147(3)	96.4

[1655] = 1+x,y,z

[2567.] = -x,1-y,2-z

DUZNUI

http://dx.doi.org/10.5517/ccvjqtw

Donor --- H....Acceptor	ARU	D – H [Å]	H...A [Å]	D...A [Å]	D - H...A [°]	A..H..A* [°]
O2 --H2 ..H1B1	4546	0.89(3)	1.76(4)	2.62(2)	162(3)	
C12 --H12B ..O1		0.99	2.52	2.868(3)	101	

[4546] = -x,-1/2+y,3/2-z

WANTAI

http://dx.doi.org/10.1055/s-0031-1289727

Donor --- H....Acceptor	ARU	D – H [Å]	H...A [Å]	D...A [Å]	D - H...A [°]	A..H..A* [°]
N --H(1N) ..O	1655	0.97(2)	1.93(2)	2.892(2)	167.4(18)	
O --H(1O) ..H(2B)	2747	0.88(3)	1.81(4)	2.68(2)	173(3)	
N --H(2N) ..O		0.96()	2.33(3)	2.838(2)	112(2)	

[2747] = 2-x,-1/2+y,2-z

[1655] = 1+x,y,z
